# Theoretical Study of the Multiferroic Properties of Pure and Ion-Doped Pb_5_*M*_3_F_19_, *M* = Fe, Cr, Al

**DOI:** 10.3390/ma17184476

**Published:** 2024-09-12

**Authors:** Iliana N. Apostolova, Angel T. Apostolov, Julia M. Wesselinowa

**Affiliations:** 1 Faculty of Forest Industry, University of Forestry, 1756 Sofia, Bulgaria; inaapos@abv.bg; 2 Department of Physics, Faculty of Hydrotechnics, University of Architecture, Civil Engineering and Geodesy, 1046 Sofia, Bulgaria; angelapos@abv.bg; 3 Faculty of Physics, Sofia University “St. Kliment Ohridski”, J. Bouchier Blvd. 5, 1164 Sofia, Bulgaria

**Keywords:** Pb_5_*M*_3_F_19_, *M* = Fe, Cr, Al, magnetization, polarization, magnetodielectric coefficient, microscopic model

## Abstract

In a first theoretical investigation of the multiferroic properties of ${\mathrm{Pb}}_{5}$${\mathrm{Fe}}_{3}$$F_{19}$ (PFF) and ${\mathrm{Pb}}_{5}$${\mathrm{Cr}}_{3}$$F_{19}$ (PCF), we analyze their magnetic, ferroelectric, and dielectric characteristics as functions of temperature, magnetic field, and ion doping concentration using a microscopic model and Green’s function theory. The temperature-dependent polarization in PFF and PCF shows a distinctive kink at the magnetic Neel temperature $T_{N}$, which vanishes when an external magnetic field is applied, indicating the multiferroic behavior of these two compounds. Ion doping effectively tunes the properties of PFF and PCF. In PFF, Cr ion doping leads to a decrease in the Neel temperature $T_{N}$, while Cr and Al ion doping lowers the ferroelectric Curie temperature $T_{C}$. In the case of PCF, we observe the enhancement of $T_{C}$ by Fe ion doping and the reduction by Al ion doping. The last result coincides well quantitatively with the experimental data. Additionally, the magnetodielectric coefficient of PFF is enhanced with the increasing magnetic field.

## 1. Introduction

Multiferroics are materials about which, in the last 10–15 years, there has been an increasing interest regarding the experimental and theoretical investigations of various spin-ordered systems wherein one phase magnetic and one ferroelectric arrangement can exist simultaneously [1,2]. At the present time, they are under intensive study in light of their many novel applications as well as new physics. The simultaneous presence of ferroelectricity and magnetism in multiferroics breaks both spatial inversion and time reversal symmetries at the macroscopic scale, which leads to many interesting phenomena and resembles the violation of these symmetries in particle physics. The symmetry-breaking in multiferroics occurs spontaneously at phase transitions rather than at the level of fundamental interactions and thus can be controlled. Depending on the origin of electric polarization, multiferroic materials are divided into two groups: type-I multiferroics, in which the magnetoelectric coupling is quadratic in the order parameters and in which the ferroelectric ordering temperature is much higher than the magnetic one (for example, ${\mathrm{BiFeO}}_{3}$, hexagonal ${\mathrm{RMnO}}_{3}$); and type-II multiferroics, in which the magnetoelectric coupling is linear and the electric polarization is induced by ordered magnetic spins (for example, orthorhombic ${\mathrm{RMnO}}_{3}$). The simultaneous breaking of inversion symmetry and time reversal symmetry can lead to the linear magnetoelectric effect and to the magnetically induced electric polarization in type-II multiferroics. These two phenomena have common microscopic origins, but they require different kinds of magnetic order.

The fluoride family containing 3*d* transition metal ions, $A_{5}M_{3}$$F_{19}$ (where *A* = Pb, Ba, Sr and *M* = Ti, V, Cr, Fe), comprises typical ferroelectric materials with a ferroelectric ordering temperature $T_{C}$ significantly higher than room temperature [3]. Ravez [4] reported the ferroelectric Curie temperatures $T_{C}$ of the ${\mathrm{Pb}}_{5}$(Cr,Ti,V,Fe${)}_{3}$$F_{19}$ family. At 295 K, ${\mathrm{Pb}}_{5}$${\mathrm{Cr}}_{3}$$F_{19}$ (PCF) crystallizes in the tetragonal I4cm space group [5,6]. ${\mathrm{Pb}}_{5}$${\mathrm{Fe}}_{3}$$F_{19}$ (PFF) is isostructural with PCF, featuring a ferroelectric Curie temperature of 725 K [4,5] and a tetragonal structure [7]. Studies of the atomic positions in the polar crystal structure of PCF (4 mm point group) indicate that the ${\mathrm{Cr}}^{3+}$ ions are displaced from the center of their octahedral sites. The Curie temperature for PCF at $T_{C}$ = 545 K is of the first-order type [4], characterized by an abrupt change in the birefringence sign at $T_{C}$. Ravez et al. [6] observed the $T_{C}$ values for the isomorphous compounds of displacive type ${\mathrm{Pb}}_{5}M_{3}$$F_{19}$ (*M* = Al, Ti, V, Cr, Fe, Ga). The compound ${\mathrm{Pb}}_{5}$${\mathrm{Al}}_{3}$$F_{19}$ (PAF) shows only ferroelectric properties because the ${\mathrm{Al}}^{3+}$ ion is nonmagnetic [8,9]. This compound is not multiferroic.

Let us note that the magnetic properties of these materials from the fluoride family, $A_{5}M_{3}$$F_{19}$, which are the reason for their multiferroic nature, were discovered many years later and are not fully clarified to this day. The spontaneous polarization has been primarily attributed to the displacement of *M* ions along the polar *c*-axis [5], i.e., the *M* ions are the origin for both the magnetism and ferroelectricity of PCF and PFF. Therefore, it can be assumed that there is a strong magnetoelectric coupling in these compounds. Blinc et al. [10] were the first to report the observation of a magnetic phase transition in multiferroic PCF. The electron paramagnetic resonance (EPR) spectra exhibit two significant anomalies: one at the ferroelectric transition temperature $T_{C}$ = 545 K and another at the magnetic transition temperature $T_{N1}$≈ 11 K. The compound also shows an antiferromagnetic transition at low temperatures of about $T_{N2}$ = 0.1 K [3]. The peaks observed at $T_{C}$ in both the EPR line width and the *g*-factor further indicate strong magnetoelectric coupling in PCF [10]. Recently, Xu et al. [7] observed similar behavior in the isostructural PFF compound. They reported two antiferromagnetic transitions at $T_{N1}$ = 78 K and $T_{N2}$ = 21 K, which might be due to different exchange interactions between the ${\mathrm{Fe}}^{3+}$ ions. The magnetoelectric coupling in PFF was confirmed by Xu et al. [7] through the observation of the magnetodielectric effect, which is attributed to the strong coupling between magnetic and structural properties.

In this paper, we investigate for the first the multiferroic properties of pure and ion-doped PFF, PCF, and PAF using a microscopic model and Green’s function theory. These materials have interesting magnetic, electric, and dielectric properties, i.e., most are multiferroics. We want to explain at a microscopic level the reason for the appearance of multiferroism in some of them as well as its tuning after ion doping, a study that has not been conducted until now.

## 2. Model and Method

The Hamiltonian that characterizes the multiferroic properties of PFF or PCF is as follows:

(1) $$H=H_{m}+H_{e}+H_{me}.$$

Here, $H_{m}$ is the Heisenberg model of the transition metal ions:

(2) $$\begin{array}{ccc} H_{m} & = & -\frac{1}{2}\sum_{ij}\left(1-x\right)J_{ij}S_{i}\cdot S_{j}-\frac{1}{2}\sum_{ij}xJ_{ij}^{Fe(Cr)-DI}S_{i}\cdot S_{j}^{DI}-\sum_{i}D_{i}{\left(S_{i}^{z}\right)}^{2}\\ & - & g\mu_{B}h\sum_{i}S_{i}^{z}. \end{array}$$

Here, $S_{i}$ represents the Heisenberg spin operator for the ${\mathrm{Fe}}^{3+}$ or ${\mathrm{Cr}}^{3+}$ ion, with $S^{z}$ denoting its *z*-component at site *i*. The parameter J<0 signifies the antiferromagnetic exchange interaction between ${\mathrm{Fe}}^{3+}$ or ${\mathrm{Cr}}^{3+}$ ions, while $J_{ij}^{Fe(Cr)-DI}$ describes the interaction between the Fe(Cr) and DI ions. The variable *x* indicates the ion doping concentration, and DI refers to the doping ion. The parameter $D_{i}>0$ represents the single-ion anisotropy, and *h* denotes an external magnetic field. It is important to note that the interaction between Fe and Cr ions induced by the doping is a positive double exchange interaction.

The ferroelectric properties of these compounds arising from the displacements of $M^{3+}$ ions along the polar *c*-axis can be modeled using the Ising model in a transverse field (TIM). Ravez [4] has shown that the ferroelectric phase transition for PCF is of the first-order type. Therefore, we must take into account, in addition to the two-spin interactions $J^{\prime }$, the four-spin interactions $J^{\prime \prime }$ [11]:

(3) $$H_{e}=-\Omega \sum_{i}B_{i}^{x}-\frac{1}{2}\sum_{ij}\left(1-x\right)J_{ij}^{\prime }B_{i}^{z}B_{j}^{z}-\frac{1}{4}\sum_{ijkl}\left(1-x\right)J_{ijkl}^{\prime \prime }B_{i}^{z}B_{j}^{z}B_{k}^{z}B_{l}^{z}.$$

Here, $B_{i}^{x}$ and $B_{i}^{z}$ are pseudo-spin operators, and Ω represents the tunneling frequency. We choose a new coordinate system by rotating the original one used in (3) by an angle θ in the xz-plane determined by the condition $\langle B^{x^{\prime }}\rangle =0$ in the new coordinate system. It is important to highlight that Blinc and de Gennes [12] proposed the TIM for describing the order–disorder behavior in ${\mathrm{KH}}_{2}$${\mathrm{PO}}_{4}$ (KDP)-type ferroelectrics. Additionally, this model has been extended to describe displacive-type ferroelectrics such as ${\mathrm{BaTiO}}_{3}$ (BTO) [13].

The magnetoelectric term that couples the two subsystems (2) and (3) can be expressed as:

(4) $$H_{me}=-g\sum_{ijkl}B_{i}^{z}B_{j}^{z}S_{k}\cdot S_{l}.$$

We consider a quadratic magnetoelectric coupling *g* between the magnetic and electric order parameters due to the significant difference between the ferroelectric Curie temperature $T_{C}$ and the magnetic phase transition temperature $T_{N}$, with $T_{C}>>T_{N}$. Additionally, Trontelj et al. [14] determined that the coupling between electric polarization and magnetization is quadratic. Utilizing the TIM and biquadratic coupling between the pseudo-spins and magnetic moments suggests that the mechanisms driving magnetic and ferroelectric orderings are independent. Consequently, this typically results in distinct transition temperatures for the two subsystems. This model has been effectively applied to other multiferroic materials where $T_{C}>>T_{N}$, such as hexagonal ${\mathrm{RMnO}}_{3}$ and ${\mathrm{BiFeO}}_{3}$ [15,16].

The magnetization *M* for a general spin *S* is defined by:

(5) $$M=\langle S^{z}\rangle =\frac{1}{N^{2}}\sum_{ij}\left[\left(S+0.5\right)\coth\left[\left(S+0.5\right)\beta E_{mij}\right]-0.5\coth\left(0.5\beta E_{mij}\right)\right],$$

where β = 1/$k_{B}$T, $k_{B}$ is the Boltzmann constant, and $E_{mij}$ is the spin wave energy calculated from the Green function $g_{ij}\left(t\right)=\ll S_{i}^{+}\left(t\right);S_{j}^{-}\gg$ using a method proposed by Tserkovnikov [17]:

(6) $$E_{mij}=\frac{\langle \left[\left[S_{i}^{+},H\right];S_{j}^{-}\right]\rangle }{\langle \left[S_{i}^{+};S_{j}^{-}\right]\rangle }.$$

This method extends beyond the random phase approximation by incorporating all correlation functions. Furthermore, it facilitates the computation of the imaginary component of the Green function.

The spontaneous polarization *P* can be derived from the Green function as follows:

(7) $${\tilde{g}}_{ij}=\ll B_{i}^{+};B_{j}^{-}\gg$$

as

(8) $$P=\frac{1}{2N^{2}}\sum_{ij}\tanh\frac{E_{fij}}{2k_{B}T}.$$

Here, $E_{fij}$ is the pseudo-spin wave energy.

In our approach, the magnetoelectric coupling *g* modifies the exchange interaction constants for spins and pseudo-spins between next-nearest neighbors.

(9) $$J_{eff}=J+2gP^{2}\cos^{2}\theta ,$$

(10) $$J_{eff}^{\prime }=J^{\prime }+2g\left(\langle S^{-}S^{+}\rangle +\langle S^{z}S^{z}\rangle \right)/\cos\theta$$

The dielectric constant ϵ(E) is observed from the following equation [18]:

(11) $$\left({\left(\Lambda /\left(\epsilon \left(E\right)-1\right)\right)}_{\alpha \beta }+\Lambda \right)G^{\alpha \beta }\left(E\right)=\delta_{\alpha \gamma };\;\;\Lambda =4\pi Z^{2}/v,$$

where *Z* is the electron charge, and *v* is the volume.

To analyze the dielectric function ϵ(k,E), we calculated the longitudinal anticommutator Green function $G^{zz}$:

(12) $$G^{zz}\left(k,E\right)=\frac{2\langle B^{z}\left(k\right)B^{z}\left(-k\right)\rangle \left(E^{2}-{\left(E_{f}\left(k\right)\right)}^{2}+2iE\gamma^{11}\right)}{\left(E^{2}-{\left(E_{f}\left(k\right)\right)}^{2}+2iE\gamma^{11}\right)\left(E+i\gamma^{33}\right)-E{\left(\epsilon^{13}\right)}^{2}}.$$

Here, $E_{f}\left(k\right)$ and $\gamma^{11}$ represent the transverse pseudo-spin wave energy and its damping, respectively, while $\gamma^{33}$ denotes the longitudinal damping. The term $\epsilon^{13}$ characterizes the coupling between longitudinal and transverse modes. To determine $\epsilon^{\prime }$ and $\epsilon^{\prime \prime }$, it is necessary to evaluate the real and imaginary parts of the Green function (12). From Equation (11), we can then derive the dielectric function ϵ(k,E), which can be analyzed for various parameters.

The magnetodielectric coefficient MC(%) is defined as:

(13) MC(%)=[ϵ(h)−ϵ(h=0)]/ϵ(h=0)%.

All equations must be calculated self-consistently.

## 3. Numerical Results and Discussion

We now present and analyze our numerical results. The calculations were carried out using programs developed in the Java programming language. A self-consistent iterative method was employed, with the model parameters specified below serving as input for the initial iteration. For each subsequent iteration, the results from the preceding calculation were used as input. The iterative process continued until the difference between consecutive iterations was less than a specified, sufficiently small value.

The numerical calculations employed the following model parameters:

1. PFF: $J^{Fe-Fe}$ = −6.69 K, $D^{Fe}$ = 0.9 K, $S^{Fe}$ = 5/2, $J^{\prime }$ = 777.41 K, $J^{\prime \prime }$ = 68.72 K, Ω = 21.19 K, *g* = 6 K, $T_{N}$ = 78 K, $T_{C}$ = 725 K.
2. PCF: $J^{Fe-Fe}$ = −6.69 K, $J^{Cr-Cr}$ = −2.21 K, $J^{Fe-Cr}$ = 4.38 K, $D^{Cr}$ = 0.93 K, $S^{Cr}$ = 3/2, $J^{\prime }$ = 584.40 K, $J^{\prime \prime }$ = 53.48 K, Ω = 32.23 K, *g* = 6 K, $T_{N}$ = 11 K, $T_{C}$ = 545 K.
3. PAF: $J^{Fe-Fe}$ = −6.69 K, $J^{Al-Al}$ = 0 K, $J^{Fe-Al}$ = 0 K, $D^{Fe}$ = 0.9 K, $S^{\mathrm{Al}}$ = 0, $J^{\prime }$ = 305.60 K, $J^{\prime \prime }$ = 27.97 K, Ω = 59.43 K, *g* = 6 K, $T_{N}$ = 0 K, $T_{C}$ = 285 K.

We will briefly outline the procedure for obtaining these model parameters. The values for the spin and pseudo-spin exchange interaction constants are estimated using the mean field theory expression $J=3k_{B}T_{C}/zS\left(S+1\right)$ below the phase transition temperatures [19], where *z* represents the number of nearest neighbors, *S* denotes the spin value, $T_{C}$ is the critical temperature, and $k_{B}$ is the Boltzmann constant. The tunneling frequency Ω is determined from the relation 2Ω = $E_{f}$ (ferroelectric energy) at very high temperatures. Using these relations, we have derived the exchange interaction constants for the materials.

### 3.1. Temperature Dependence of the Magnetization and Polarization of PFF, PCF, and PLF

**Figure 1:**
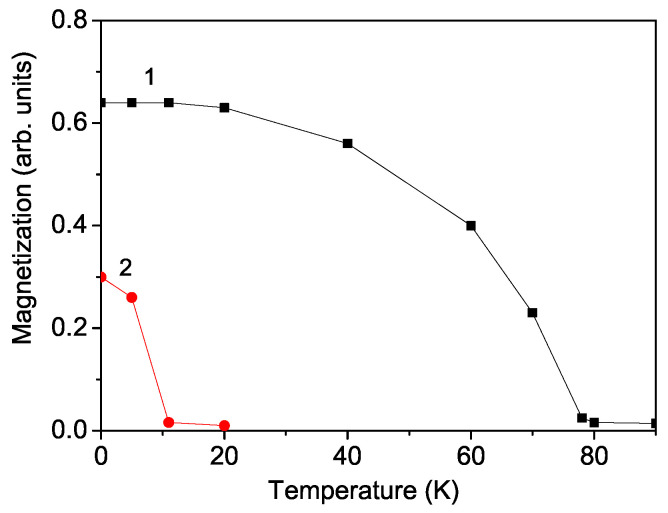
Temperature dependence of the magnetization *M* for PFF (curve 1) and PCF (curve 2) for *h* = 100 Oe. (Color online).

Let us emphasize that the magnetic properties of PFF, PCF, and PLF, due to the magnetoelectric interaction, can be manipulated through an external electric field and vice versa; the ferroelectric ones can be changed through an external magnetic field. Firstly, we will investigate the multiferroic properties of pure PFF and PCF. They are antiferromagnetic compounds with negative exchange spin interaction (J<0). Therefore, the magnetization *M* is zero. However, when applying an external magnetic field *h*, there appears to be a small magnetization *M*. The observed magnetism may arise from the magnetoelectric coupling between the ferroelectric polarization and the magnetization, which induces spin canting in the antiferromagnetic interaction among neighboring ${\mathrm{Fe}}^{3+}$ ions. Figure 1 displays the temperature dependence of the magnetization *M* for *h* = 100 Oe in pure PFF and PCF, shown as curves 1 and 2, respectively. It is evident that the magnetization *M* decreases as the temperature *T* increases. The Neel temperatures $T_{N}$ for PFF and PCF are approximately 78 K and 11 K, respectively, which coincide with the experimental data [7,10].

**Figure 2:**
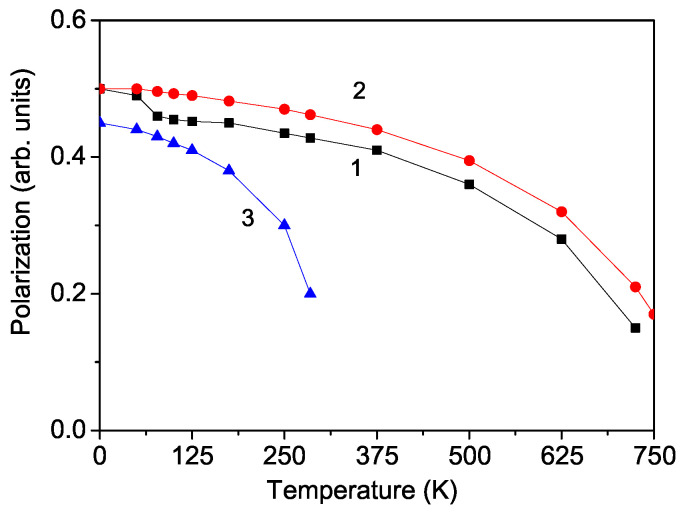
Temperature dependence of the polarization *P* in PFF for different magnetic field *h* values: 0 (curve 1), 100 Oe (curve 2), and PLF (curve 3).

The $A_{5}M_{3}$$F_{19}$ family contains typical ferroelectric materials, where the ferroelectric properties arise from the displacement of $M^{3+}$ ions along the polar direction from the centers of their corner-sharing ${\mathrm{MF}}_{6}$ octahedra. Smaller $M^{3+}$ ions lead to greater displacements, suggesting that the Curie temperature $T_{C}$ should increase as the ionic radius decreases when moving from *M* = Al, Cr, Fe. Figure 2 shows the polarization *P* as function of temperature *T*. In PFF, a first-order phase transition is observed around $T_{C}$ = 725 K, where *P* remains finite (curve 1). Ravez [4] reported similar polarization behavior for PCF. Let us emphasize that, around $T_{N}$ = 78 K, we observe a kink or an anomaly, which is evidence for the multiferroic character of PFF. Moreover, applying an external magnetic field *h* causes an increase in polarization *P* and eliminates the kink (curve 2). The changes in the polarization caused by an external magnetic field *h* are a typical property of a multiferroic material. In PCF, a kink for the polarization *P* at $T_{N}$ = 11 K is also obtained. Unfortunately, experimental data for this kink are lacking for both compounds. Ravez [4] has studied the electrical properties of PCF in the temperature interval between 300 and 600 K.

PAF shows only ferroelectric properties (see Figure 2, curve 3). It is not multiferroic because the Al ion is nonmagnetic. Therefore, there is no kink in P(T).

### 3.2. Ion-Doping Dependence of the Neel Temperature

Doping with ions of varying ionic radii compared to the host ions introduces different strains, which alter the exchange interaction constants represented by *J*. These constants are inversely related to the distance between spins and the lattice parameters. In doped states, represented as $J_{d}$ and $J_{d}^{\prime }$, the exchange interaction constants may be either increased or decreased compared to those in the undoped states *J* and $J^{\prime }$, depending on whether the strain is compressive or tensile. These adjustments allow for the fine-tuning of the compound’s properties.

**Figure 3:**
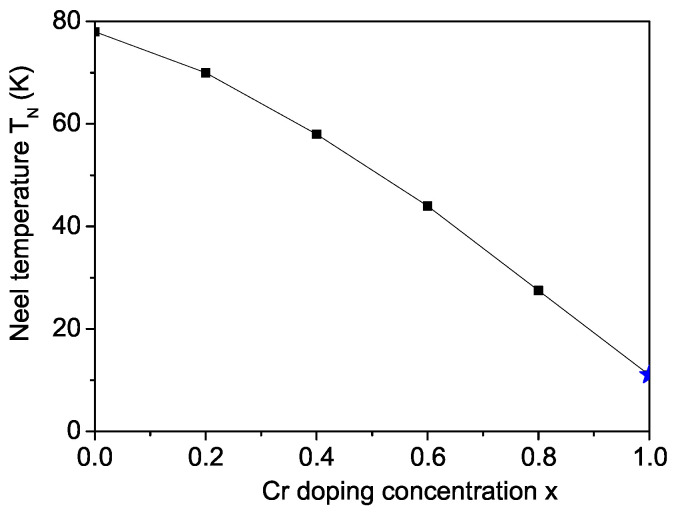
Doping concentration dependence of the Neel temperature $T_{N}$ in Cr-doped PFF. For *x* = 1, i.e., for PCF, we obtain the Neel temperature of 11 K, denoted as a blue star, which is reported by Blinc et al. [10]. This shows that our results observed with the proposed model and method are correct.

Doping PFF with the ${\mathrm{Cr}}^{3+}$ ion (*r* = 0.755 Å), whose radius is larger than that of the host ${\mathrm{Fe}}^{3+}$ ion (*r* = 0.69 Å), induces a tensile strain. This tensile strain leads to a reduction in the exchange interaction constants in the doped states compared to those in the undoped states, resulting in $J_{d}<J$ and $J_{d}^{\prime }<J^{\prime }$. As a result, the magnetization decreases with increasing Cr doping concentration *x*. Figure 3 displays the variation of the Neel temperature $T_{N}$ in Cr-doped PFF as a function of *x* for $J_{d}=0.8J$ and $J_{d}^{\prime }=0.8J^{\prime }$. It is observed that $T_{N}$ decreases with an increasing Cr doping concentration *x*. Let us emphasize that, for *x* = 1, i.e., for PCF, we obtain the Neel temperature of 11 K, which is reported by Blinc et al. [10]. This shows that our results observed with the proposed model and method are correct.

### 3.3. Ion-Doping Dependence of the Curie Temperature

Controlling the Curie temperature through different ion doping techniques is anticipated to optimize properties such as the linear electro-optic coefficients and dielectric permittivity, which often peak near $T_{C}$. Therefore, as a next step, we will study the Curie temperature $T_{C}$ of Cr- and Al-doped PFF.

**Figure 4:**
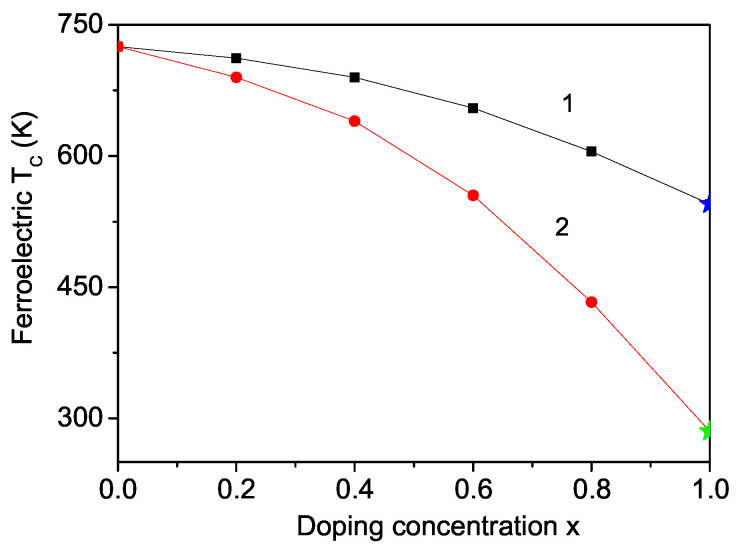
Doping concentration dependence of the ferroelectric Curie temperature $T_{C}$ in PFF for different doping ions: Cr (1), and Al (2). The blue and green stars are the experimental data of [10] and [4], respectively. (Color online).

The ionic radii of the doping ions ${\mathrm{Cr}}^{3+}$ (0.756 Å) and ${\mathrm{Al}}^{3+}$ (0.775 Å) are larger than that of the host ${\mathrm{Fe}}^{3+}$ ion (0.64 Å). This difference induces a tensile strain, resulting in a decrease in the Curie temperature $T_{C}$ with increasing doping ion concentration *x*. Figure 4 presents the results, with curves 1 (for $J_{d}=0.8J$) and 2 (for $J_{d}=0.7J$). The $T_{C}$ values for *x* = 1 are in good quantitative agreement with the experimental data for PCF ($T_{C}$ = 545 K [10]) and PAF ($T_{C}$ = 285 K [4]).

**Figure 5:**
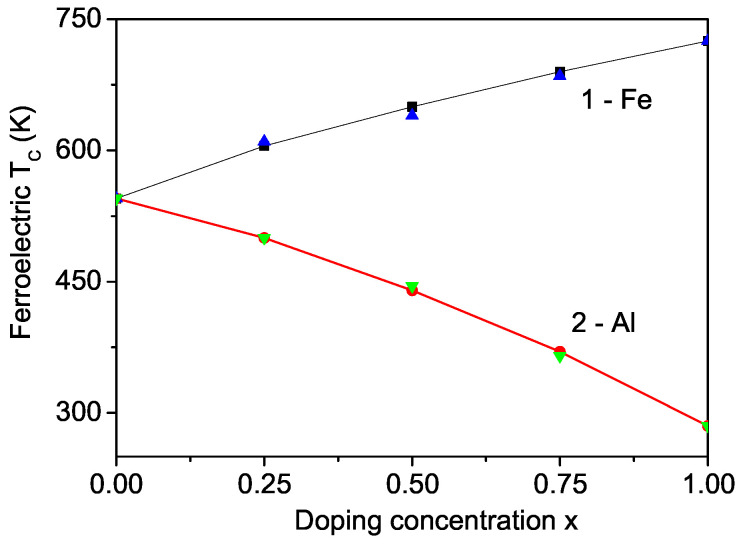
Doping concentration dependence of the ferroelectric Curie temperature $T_{C}$ in PCF for different doping ions: Fe (1), and Al (2). The blue and green triangles are the experimental values from Ref. [20]. (Color online).

Doping PCF with different ions also allows for the tuning of its electrical properties. Specifically, substituting the Cr ion with an Fe ion results in an increased average atomic displacement from the para-electric position at the center of the fluorine atom octahedron, whereas substituting with an Al ion leads to a decreased average atomic displacement. Consequently, Fe doping enhances the polarization *P* compared to Al doping. Figure 5 displays the Curie temperature $T_{C}$ for Fe- and Al-doped PCF. In this compound, where the ionic radius of the Cr ion is larger than that of Fe and smaller than that of Al, an increase in $T_{C}$ is observed with Fe doping (curve 1, $J_{d}=1.2J$), while a decrease occurs with Al doping (curve 2, $J_{d}=0.8J$). This behavior is consistent with the experimental data reported by Ravez et al. [20] and Andriamampianina et al. [21]. Furthermore, the results for the Al doping shown in Figure 5 are in good quantitative agreement with the experimental findings of Ravez et al. [20], confirming that our model and approximation are effective in explaining the multiferroic properties of both pure and ion-doped PCF.

### 3.4. Magnetodielectric Coefficient for Pure PFF

**Figure 6:**
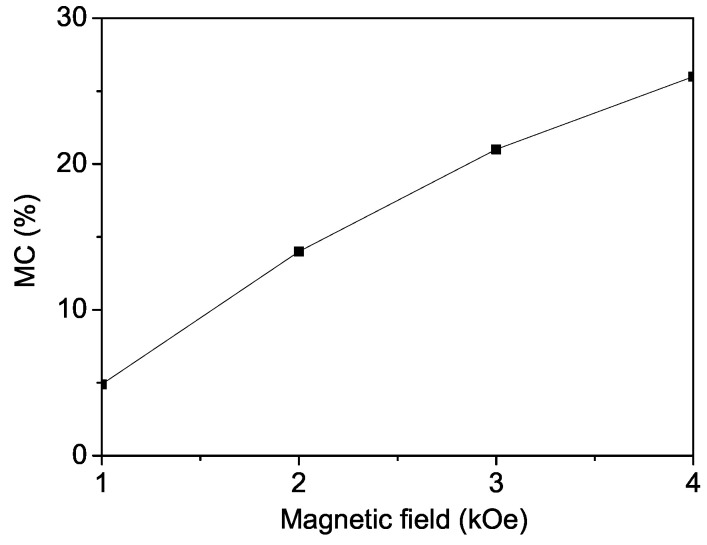
Magnetic field dependence of the magnetodielectric coefficient MC(%) in PFF.

We also investigated the real part of the dielectric constant ϵ for pure PFF, in both the presence and absence of an external magnetic field *h*. From Equation (13), we computed the magnetic field dependence of the magnetodielectric coefficient MC. The results, shown in Figure 6, reveal that MC increases with the increasing magnetic field *h*, which aligns with the experimental findings of Xu et al. [7]. This increase in MC confirms the magnetoelectric coupling attributed to the strong interaction between magnetic and structural effects.

## 4. Conclusions

Using a microscopic model and Green’s function theory, we have theoretically investigated the multiferroic properties of ${\mathrm{Pb}}_{5}$${\mathrm{Fe}}_{3}$$F_{19}$ (PFF) and ${\mathrm{Pb}}_{5}$${\mathrm{Cr}}_{3}$$F_{19}$ (PCF) for the first time. Our study covers the dependence of the magnetic, electric, and dielectric properties of these materials on temperature, magnetic field, and ion doping. We observed a kink in the polarization versus temperature curve for PFF and PCF at the magnetic Neel temperature $T_{N}$, which disappears when an external magnetic field is applied, providing evidence of their multiferroic behavior. The effects of doping are also studied. The polarization of PAF P(T) does not exhibit a peak because PAF is not multiferroic. The Al ion is nonmagnetic. The Neel temperature $T_{N}$ decreases in Cr-doped PFF. Doping with Cr and Al ions can reduce the ferroelectric Curie temperature $T_{C}$ in PFF. For PCF, we observe enhancing of $T_{C}$ by Fe ion doping and reducing by Al ion doping. The recent findings show a good quantitative agreement with the experimental data from Ravez et al. [4], confirming the accuracy of our proposed model and method in explaining the multiferroic properties of both pure and doped materials. To further validate the presence of magnetoelectric coupling in PFF, we conducted magnetodielectric studies. Additionally, we evaluated the dielectric constant ϵ of pure PFF. The observed increase in the magnetodielectric coefficient MC with an increasing magnetic field *h* further supports the multiferroic behavior of PFF.

Recently, Zhang et al. [22] reported on the magnetic properties of the multiferroic compound ${\mathrm{Ba}}_{5}$${\mathrm{Fe}}_{3}$$F_{19}$. Earlier, Ravez et al. [23] examined the ferroelectric properties and phase transitions of $A_{5}$$M_{3}$$F_{19}$ compounds, with A = Sr, Ba and M = Ti, V, Cr, Fe, Ga. They found that substituting Pb with Sr or Ba increases the ferroelectric phase transition temperature $T_{C}$, which ranges between 700 K and 1100 K. Let us emphasize that our model and method could also describe the properties of other compounds of the fluoride family $A_{5}$$M_{3}$$F_{19}$ having the same structure and similar properties, for example, ${\mathrm{BaFe}}_{3}$$F_{19}$. Therefore, a theoretical investigation of the multiferroic behavior of these materials is planned for a future study.

Moreover, we hope that our theoretical results of the observed kink at $T_{C}$ in the temperature dependence of the polarization in PFF and PCF, but not in PLF, will be confirmed by experimental data in the future. 

## Data Availability

Data sharing is not applicable to this article, as no datasets were generated or analyzed during the current study.
